# Integrating the Gender Perspective into Literature Studies to Enhance Medical University Students’ Gender Awareness and Critical Thinking

**DOI:** 10.3390/ijerph17249245

**Published:** 2020-12-10

**Authors:** Hung-Chang Liao, Ya-huei Wang

**Affiliations:** 1 Department of Health Services Administration, Chung Shan Medical University, Taichung 40201, Taiwan; hcliao@csmu.edu.tw; 2 Department of Medical Education, Chung Shan Medical University Hospital, Taichung 40201, Taiwan; 3 Department of Applied Foreign Languages, Chung Shan Medical University, Taichung 40201, Taiwan

**Keywords:** gender perspective, literature study, gender awareness, gender consciousness, critical thinking

## Abstract

Objective: This study attempted to integrate the gender perspective into literature studies to allow medical university students to examine internalized gender prescriptions and investigate whether the integration of the gender perspective into literature studies would create any difference among students in gender awareness and critical thinking. Methods: This study used fifteen-week quasi-experimental research to verify the feasibility of using the gender perspective in literature studies to arouse medical university students’ gender awareness and critical thinking. Before and after the intervention, a gender awareness test and a critical thinking disposition test were carried out by both the experimental group (41 students) and control group (41 students). Results: The findings show that regarding gender awareness, with the integration of the gender perspective into literature studies, medical university students had significantly higher post-test scores for “public gender consciousness” and “private gender consciousness.” In regard to critical thinking, they also had significantly better post-test scores in “systematicity and analyticity,” “maturity and skepticism,” and “inquisitiveness and conversance.” Conclusion: This study demonstrated that the integration of the gender perspective into literature studies could result in positive learning outcomes among medical university students in terms of gender awareness and critical thinking.

## 1. Introduction

In issues concerning women and men, the terms “gender” and “sex” must be examined. The term “sex” usually refers to the biological categories of male and female, primarily based on reproductive potential, while “gender” refers to the social meaning associated with male or female [1]. Thinking of gender as the result of nurture and sex as the result of nature [2], the interwoven connection between sex and gender allows people, either consciously or unconsciously, to incorporate male and female gender archetypes into their internalized gender prescriptions, to meet others’ expectations, and to govern their expectations of others [3], neglecting the notion that gender is the result of a social construction.

Therefore, based on social role theory, both men and women have to act differently to confirm the gender stereotypes placed on them [4]. Specifically, women must exhibit traditionally feminine behaviors, such as emphasizing personal relationships, while men have to exhibit traditionally masculine behaviors, such as being assertive and being more likely than women to work outside the home [5].

Like Beauvoir’s [6] declaration that “One is not born, but rather becomes, woman,” gender is not what people are born with but how people behave in the socially constructed power structure [7]. Gender socialization suggests the idea that males should be dominant, tending to be rational, aggressive, and controlling, while females should be more emotional, submissive, and concerned about others [3,4]. Hence, the socially constructed power structure allows women to go through the process of gender socialization patterns, through which they are educated and tamed for subordination, not for equality [8]. Therefore, in order to survive in the traditional gender hierarchy, women have become accustomed to the deprivation of their autonomy, having no choice but to subordinate themselves to gender norms [9].

Critical thinking can be a useful tool for examining socially constructed gender norms, such as masculinity and femininity. The American Philosophical Association [10] included six core elements in critical thinking: interpretation, analysis, evaluation, inference, explanation, and self-regulation. Research has defined critical thinking as one’s ability to use a logic guide to independently manage one’s thought structures for one’s behavior and beliefs [11,12]. With the disposition to think critically, one would be able to analyze, synthesize, and evaluate interrelationships hidden in the knowledge construction process, as well as look for evidence to lead logical judgment [13,14]. While being stimulated appropriately, critical thinking can help develop gender awareness and, in turn, influence ones’ behaviors and attitudes toward gender roles [15]. In thinking critically, one may step back from gender issues, examine socially constructed gender norms, and question the legitimacy of men’s control over women [16]. Research has shown the importance of integrating gender competence into medical education [17,18,19]. Schiebingers and Miller [17] worked on a systematic review on gendered innovation in the field of medicine and healthcare, also illustrating Stanford University’s initial attempt to integrate sex and gender analysis the medical education to promote gender awareness and equality. Kristoffersson, Andersson, Bengs, and Hamberg [18], identifying gender stereotyping and biased treatment in medical studies and clinical training, advocated the inclusion of gender education in the formal medical curriculum in order to develop the gender competence of medical students and the faculty.

In order to increase medical university students’ awareness that the traditional male-constructed gender norms are used to discipline women in the hierarchy system, literature studies can be used to access the wealth of human experience on gender issues. Conventionally, works of literature have served as conduct books, using moral norms in the texts to pressure women to be attentive and submissive. Therefore, readers, especially women, may unconsciously follow the plots and conform to the expected gender images with appropriate feminine traits or behaviors [4,11,20].

While literature studies can be used for entertainment or as a means to introduce conduct books to discipline women in the hierarchy system [4,21], literature studies may have another function to expose readers to literary texts regarding social gender and morality [22], allowing them to step back from gender issues and to critically reflect upon socially constructed gender norms. Therefore, while following the plot, readers may identify with the oppressed characters, especially suffering females, and develop greater empathy toward those suffering [23]. In the study, instead of using literature texts that present social norms as a tool to construct medical university students’ gender identities, the researchers intended for literature texts to be interpreted from the gender perspective to allow students to examine their internalized gender prescriptions and become more aware of gender archetypes, hence reflecting on the traditional gender role expectations of others. By doing so, the study further examined whether the integration of the gender perspective into literature studies would create any difference between the students in terms of gender awareness and critical thinking. Hence, the study proposed the following research hypotheses:

**Hypothesis 1 (H1).** *Medical university students exposed to the integration of the gender perspective into literature studies would have greater gender awareness than those not receiving such exposure.*

**Hypothesis 2 (H2).** *Medical university students exposed to the integration of the gender perspective into literature studies would have a higher level of critical thinking than those not receiving such exposure.*

## 2. Material and Methods

### 2.1. Experimental Design and Procedure

The study used quasi-experimental research to verify the possibility of using the gender perspective in literature studies because there was no way to randomly assign students to the experimental and control groups. The study included eighty-two medical university students. Before the intervention, both groups completed the Critical Thinking Disposition Assessment (CTDA) and the Gender Awareness Scale to test the homogeneity of the two groups in the two measures. 

The same instructor taught the two groups, using the same literature works and evaluation procedure. However, the control group (41 students) read literature works for pleasure, analyzing the literature texts following the traditional methods, mainly grammar, translation, and summary; however, the gender ideology rooted in the literature works was somewhat unconsciously implanted in the students. The experimental group (41 students), while undertaking the literature study, consciously used the gender perspective to question and reflect on the traditional and socially constructed gender norm, such as the patriarchal system, gender politics, and ideology, and further examined whether the social norms acted as a mechanism to justify men’s control over women. To facilitate students’ literature discussion or reflection from the gender perspective, at the beginning of the study, the instructor introduced gender-related terms such as sex/gender, gender discrimination/equality, masculinity/femininity, feminist psychology, empowerment, liberation, etc. Therefore, while analyzing the characters and events in the literature study, the students were able to examine the artificiality of the gender binary and realized that people are not born feminine or masculine but are forced into gender stereotypes. The intervention lasted for fifteen weeks, two periods a week, plus self-study and e-discussion. Moreover, both the experimental and control groups had to interact online with their group-mates on an e-learning website (http://lms.csmu.edu.tw/course/42386) [24]. They were required to post their discussion or reflection upon the proposed topics based on the literature study (see Table 1). With the acquisition of gender terms and gender perspective training, the experimental group students might reflect on the proposed issues from the gender perspective, while control group students might express their ideas based on the conventional conceptualization of gender norms. Table 1 contains the course schedule. 

After the fifteen-week intervention, the same measures, used as the evaluation criteria, were administered to both groups to examine the feasibility of integrating the gender perspective into literature studies in terms of gender awareness and critical thinking.

### 2.2. Measures, Validity, and Reliability

#### 2.2.1. Gender Awareness Scale 

To measure students’ levels of gender awareness, a Chinese version of the Gender Awareness Scale, mainly based on Snell and Johnson’s Multidimensional Gender Consciousness Questionnaire [31], was used to access students’ gender awareness. The MGCO is a 30-item, five-point Likert scale used to assess both women’s and men’s public gender consciousness (11 items) and private gender consciousness (19 items), with 4 meaning “very characteristic of me” and 0 meaning “not at all characteristic of me.” Public gender consciousness refers to the degree to which an individual is conscious of how other people view his or her masculinity or femininity. Private gender consciousness refers to the degree to which an individual is conscious of his or her masculinity or femininity. Research found reliability scores for the MGCQ to be acceptable [32]. Cronbach’s alpha values for the two subscales and entire questionnaire were 0.74, 0.86, and 0.87, respectively. 

#### 2.2.2. Critical Thinking Disposition Assessment (CTDA)

The researchers used the Critical Thinking Disposition Assessment [33] to assess students’ critical thinking dispositions. The CTDA is a 19-item rated, 7-point Likert scale and includes three factors: “systematicity and analyticity” (8 items), “inquisitiveness and conversance” (6 items), and “maturity and skepticism” (5 items), with 7 meaning “strongly agree” and 1 meaning “strongly disagree.” “Systematicity and analyticity” refers to the capacity to draw conclusions by systematic thinking and logical analysis. “Inquisitiveness and conversance” refers to the capacity to inquire and explore questions in consideration of multiple perspectives. “Maturity and skepticism” refers to the capacity to raise questions and tolerate different standpoints. Cronbach’s alpha values for the three factors and entire scale were 0.91, 0.86, 0.86, and 0.94, respectively.

### 2.3. Data Analysis

The study used Statistical Packages for the Social Science (SPSS), Version 14.0 (IBM, Armonk, NY, USA) to analyze the quantitative data. One-way MANOVA (multivariate analysis of variance) and one-way MANCOVA (multivariate analysis of covariance) were used to determine whether there were any differences between the two groups, using pretest scores as covariates to control the possible initial differences. In addition, two-way MANOVA was also used to explore the differences in terms of gender awareness and critical thinking.

### 2.4. Ethical Consideration

The study was a project supported by the school (project identification code: 105-CSMU-GE-002) and was carried out following the rules of the Declaration of Helsinki [34]. To protect participants’ identities and confidentiality of their personal information, the responses were anonymous and all data were identified using numbers.

## 3. Results

**Hypothesis 1 (H1).** *Medical university students exposed to the integration of the gender perspective into literature studies would have greater gender awareness than those not receiving such exposure.*

To test Hypothesis 1 (gender awareness), using a one-way MANOVA, the pretest results (Wilks’ Λ = 0.987; *F* (2, 79)= 0.523; *p*-value = 0.595 > 0.05) showed no significant differences (*F* (1, 80) = 0.995 and 0.001; *p* = 0.321 and 0.971 > 0.05) between the means of the experimental group (*n* = 41; means (M) = 34.90 and 56.15) and the control group (*n* = 41; M = 33.22 and 56.07; see Table 2) on “public gender consciousness” and “private gender consciousness.” 

After intervention, the researchers used a one-way MANCOVA to investigate whether pretest results would make any difference in the post-test results, using pretest scores as covariates. The results revealed that in the gender awareness test, there was a statistically significant relatedness between the pretests and post-tests in both the “public gender consciousness” (Wilks’ lambda: 0.859; *p* = 0.003) and “private gender consciousness (Wilks’ lambda: 0.771; *p* < 0.000; see Table 3). 

In the adjusted post-test means of the subscales (see Table 4), the researchers found that in the “public gender consciousness” and “private gender consciousness,” the post-test means of the experimental group (means = 39.72 and 65.78) were significantly higher than the means of the control group (means = 37.25 and 61.05; *p <* 0.05). 

Further using two-way MANOVA, the researchers intended to explore which factors would affect “public gender consciousness” and “private gender consciousness,” with the pretest and post-test as the dependent variable (also as the test factor) and with the experimental group and control group as the independent variable (also as the group factor). The analysis showed that in “public gender consciousness” and “private gender consciousness,” there was no significant interaction between the test factor and the group factor (*p* > 0.05), although the test factor had a significant effect on public and private gender consciousness (*p <* 0.01; see Table 5 and Table 6).

**Hypothesis 2 (H2).** *Medical university students exposed to the integration of the gender perspective into literature studies would have a higher level of critical thinking than those not receiving such exposure.*

To test Hypothesis 2 (critical thinking), using a one-way MANOVA, the pretest results (Wilks’ Λ = 0.989; *F* (3, 61)= 0.296; *p*-value = 0.828 > 0.05) showed no significant differences (*F* (1, 80) = 0.015, 0.287, and 0.359; *p* = 0.904, 0.594, and 0.551 > 0.05) between the means of the experimental group (M = 40.17, 31.05, and 26.80) and those of the control group (M = 40.34, 30.39, and 26.17; see Table 7) in “systematicity and analyticity,” “inquisitiveness and conversance,” and “maturity and skepticism.”

After intervention, the researchers used a one-way MANCOVA to investigate whether pretest results would create any difference in post-test results, using pretest scores as covariates. The results demonstrated a significant relatedness between the pretests and post-tests in all of the subscales: “systematicity and analyticity” (Wilks’ lambda: 0.639; *p <* 0.000), “inquisitiveness and conversance” (Wilks’ lambda: 0.789; *p <* 0.000), and “maturity and skepticism” (Wilks’ lambda: 0.752; *p <* 0.000; see Table 8).

While examining the adjusted post-test means of the subscales (see Table 9), the researchers discovered that in “inquisitiveness and conversance,” the post-test means of the experimental group (means = 37.29) were significantly higher than the means of the control group (means = 34.47) at the 0.05 level. Notably, in “systematicity and analyticity” and “maturity and skepticism,” the experimental group had significantly higher post-test means (means = 48.93 and 33.14) than the control group (means = 44.73 and 30.55) at 0.01 level.

Further using two-way MANOVA, the researchers explored which factors would affect “systematicity and analyticity,” “inquisitiveness and conversance,” and “maturity and skepticism,” with the pretest and post-test as the dependent variable (also as the test factor) and with the experimental group and control group as the independent variable (also as the group factor). The analysis showed that in “inquisitiveness and conversance” and “maturity and skepticism,” there was no significant interaction between the test factor and the group factor (*p* > 0.05), although the test factor had a significant effect on these characteristics (*p* < 0.01; see Table 10 and Table 11). In addition, in “systematicity and analyticity,” there was a significant interaction between the test factor and the group factor (*p* < 0.01), and the test factor had a significant effect on “systematicity and analyticity” (*p* < 0.01; see Table 12).

## 4. Discussion

Aiming to deconstruct the unequal power relationship in the gender hierarchy system, the study integrated the gender perspective into literature studies to allow medical university students to examine their internalized gender ideology and to examine the possibility of using the gender perspective in literature studies. The results of the study revealed that the integration of the gender perspective into literature studies would positively affect students in terms of gender awareness and critical thinking.

**Figure 1:**
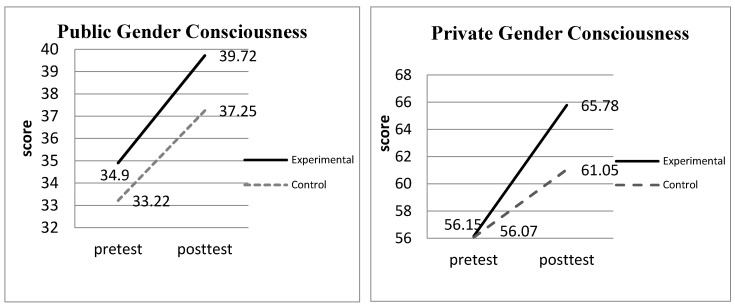
The improvements of gender awareness in the experimental and control groups.

Regarding gender awareness, medical university students exposed to the integration of the gender perspective into literature studies had significantly greater gender awareness in terms of “public gender consciousness” and “private gender consciousness.” Based on the post-test results of the Gender Awareness Scale, it can be demonstrated that following gender perspective training, experimental group students were able to read literary texts from the gender perspective regarding social gender and gender norms. Shifting to the gender lens to interpret literary texts, the students began to question whether socially constructed gender prescriptions attempt to force women into the fulfilling the expectations of others [22,35]. Furthermore, with gender consciousness, students can reflect on their own genders (bring male or male), further realizing that socially constructed gender norms attempt to force women into being submissive and nurture and men as assertive and dominating as a means to maintain the stability of the patriarchal system [4,8,9,20]. Moreover, the students that received gender perspective training and therefore became more aware of the expected gender roles were more alert to how people perceive men and women than those who did not receive such training. Furthermore, they were more aware of how others observe their own gender images and why women behave cautiously to meet gender role expectations. Hence, they were more inclined to identify the danger of crossing the gender border, realizing that those who dare to trespass the gender-prescribed border would experience the same punishment as the characters in the literary texts. The results correspond with Lindberg’s [36] study in that the integration of the gender perspective into studies can facilitate students’ discovery of what others fail to notice. It is therefore no surprise that students who used the gender perspective in the literature study exhibited higher gender awareness in terms of both public and private gender consciousness (see Figure 1).

**Figure 2:**
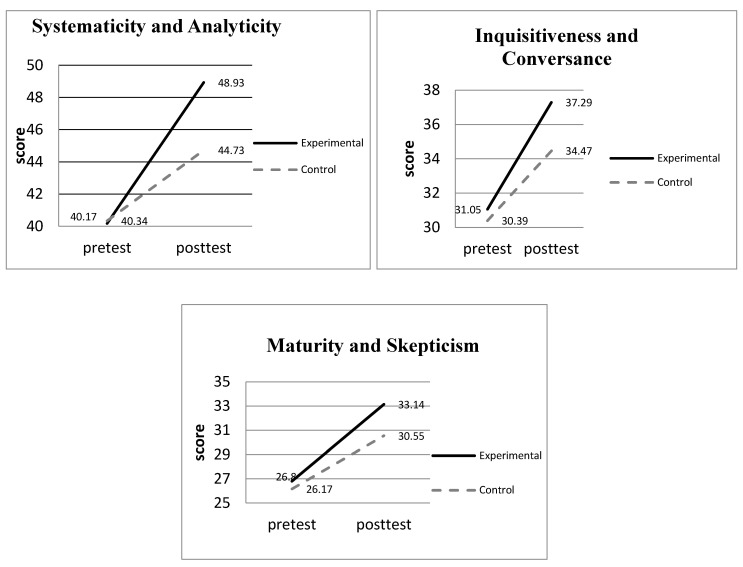
The improvements of critical thinking in the experimental and control groups.

The research results also show that the students who used the gender perspective in the literature study exhibited better critical thinking post-test scores in terms of “systematicity and analyticity,” “maturity and skepticism,” and “inquisitiveness and conversance” (see Figure 2). Based on the post-test results of the critical thinking assessment, it can be demonstrated that while reading literature from the gender perspective with critical thinking, the students may have discovered conflicting or contradictory statements regarding gender and may have begun to question why females are not treated the same way males are treated. Moreover, instead of using literature texts as social norms to confirm gender identity, the medical university students using gender perspective in the literature study were able to read between the lines while critically interpreting the literature texts from the gender perspective, and they were able to reflect on the patriarchal system as a socially constructed mechanism used to justify men’s control over women [16]. Moreover, compared to the control group students using conventional methods to read the literature texts, the experimental students consciously used the gender perspective to question and reflect on the traditional and socially constructed gender norm—they were encouraged to be inquisitive and to further examine the existing social norms. Hence, their critical thinking can be enhanced. The research results correspond with Bierema’s [37] study in that while examining gender from a new perspective, people can step back, critically examine gender ideology, and further question the legitimacy of gender norms, thus questioning socially determined gender roles. Therefore, the statistical results show that students who use the gender perspective in literature studies can foster critical thinking and raise questions about gender issues. Hence, these students’ critical thinking levels can be improved.

## 5. Conclusions

This study investigated whether the integration of the gender perspective into literature studies would create any difference between students in term of gender awareness and critical thinking. The findings showed that the participants using the gender perspective to study literature had significantly higher gender awareness, both in public and private gender consciousness, and significantly higher critical thinking levels in “systematicity and analyticity,” “maturity and skepticism,” and “inquisitiveness and conversance.”

This study demonstrated that the integration of the gender perspective into literature studies could bring about positive learning outcomes among medical university students in terms of gender awareness and critical thinking. Those exposed to the integration of the gender perspective into literature studies were able to reflect on the proposed issues from the gender perspective. Realizing the importance of integrating gender competence into medical education, future studies may use women’s literature as a means for readers to interpret women’s life stories from the gender perspective, to identify gender oppression, and to foster empathy and hence respect for others.

## Figures and Tables

**Table 1:** The course schedule.

Week	Course Schedule
1–2	“The Yellow Wallpaper” [25] Synopsis: The story follows a woman’s mental illness and her final breakdown. After the birth of her baby, her husband rents an old colonial mansion for her to relax. However, she is not allowed to work, not even writing or taking care of her baby, but is encouraged to eat and exercise. Having nothing to do, she gets bored and examines the torn yellow wallpaper. She finds that there is a figure creeping inside…. Discussion/Reflection: female hysteria; postnatal depression; women’s discourse
3–5	“The Rose for Emily” (William Faulkner, 1930) [26] Synopsis: The story follows the fall of the aristocratic Grierson family in Yoknapatawpha County, Jefferson City, Mississippi. After her father’s death, around 30 years old, Emily is totally isolated, except for the company of a black servant. Emily keeps herself away from the community until she falls into love with Homer Barron, who refuses to get married. In order to be with Homer, Emily goes to the druggist to buy…. Discussion/Reflection: women’s role in society; marriage market; mental illness; sexual object
6–7	“To Room Nineteen” (Doris Lessing, 1980) [27] Synopsis: The story describes the seemingly perfect family life of Susan and Matthew Rawlings. Being an intellectual, Susan has always been proud of being capable to make sensible decisions to keep the family harmony and take care of her four children. Even after her husband commits adultery, Susan resolves to use her intelligence to understand and forgive Matthew. However, after the youngest children begins school, Susan seems not satisfied by the seemingly perfect family and turns to …. Discussion/Reflection: gender and achievement; women’s rights; adultery; mental and physical health of women
8–10	“The Necklace” (Guy de Maupassant, 1984) [28] Synopsis: A beautiful woman, Mathilde Loisel comes from a lower-middle-class family and marries a low-paid clerk. Not satisfied with her life but fascinated with material wealth, Mathilde has always imagined herself as an aristocratic woman. One day, Mathilde attended a party with a beautiful dress and a fancy diamond necklace borrowed from a friend. Mathilde enjoyed the party. However, upon returning home, Mathilde found that the necklace…. Discussion/Reflection: marrying up; marrying down; materialism; women’s virtue; appearance or spirit
11–12	“The Story of an Hour” (Kate Chopin, 1894) [29] Synopsis: The title refers to the time between when Mrs. Louise Mallard, afflicted with “a heart trouble,” hears of her husband’s death and the time she discovers that her husband is actually not dead after all. Upon hearing the news of his death, Mrs. Mallard is very heartbroken and goes to her room alone to reflect upon her life with her husband. However, when her grief subsides, she hears an inner voice whispering…. Discussion/Reflection: ideal wife; freedom; women’s independence; heart attack
13–15	“Everyday Use” (Alice Walker, 1973) [30] Synopsis: The story follows an African-American woman, Mama, living in the rural South and her relationship with her two daughters, Maggie and Dee. Mama lives with her younger daughter Maggie, and both have followed traditional black culture. Maggie is dull, unattractive, timid, and with burned scars. In contrast to Maggie, Dee is an educated, beautiful, attractive, and stylish woman…. Discussion/Reflection: women and education; sibling rivalry; disability; mother–daughter relationship

**Table 2:** One-way multivariate analysis of variance (MANOVA) results on the gender awareness pretest.

Pretest	Group	Wilks’ Λ = 0.987 *F* (2, 79)= 0.523, *p*-Value = 0.595		
		Mean	(SD)	*F* (1, 80) (*p*-value)
Public gender consciousness	Experimental	34.90	(6.58)	0.995
	Control	33.22	(8.57)	(0.321)
Private gender consciousness	Experimental	56.15	(9.68)	0.001
	Control	56.07	(8.28)	(0.971)

Experimental group: *n* = 41; control group: *n* = 41; SD: standard deviation; *F* (1, 80): tests of between-subjects effects; Wilk’s Λ: Wilks’ lambda; *F* (2, 79): *F* (hypothesis degrees of freedom, error degrees of freedom).

**Table 3:** One-way multivariate analysis of covariance (MANCOVA) results on the gender awareness post-test.

**Post-Test**	**Covariance**	
	**Public Gender Consciousness (Pretest)**	**Private Gender Consciousness (Pretest)**
	**Wilks’ Λ**	
	***F* (2, 77)**	
	**(*p*-Value)**	
	0.859	0.771
	6.340	11.411
	(0.003)	(<0.000 **)
	**Tests of Between-Subjects Effects**	
	***F* (1, 78)**	
	**(*p*-Value)**	
Public gender consciousness	10.360	21.916
	(0.002 **)	(<0.000 **)
Private gender consciousness	0.645	0.000
	(0.424)	(0.997)

Experimental: *n* = 41; control: *n* = 41; SD: standard deviation; Wilks’ Λ: Wilks’ lambda; *F* (2, 77): *F* (hypothesis degrees of freedom, error degrees of freedom); ** *p* *<* 0.01.

**Table 4:** MANCOVA comparison of the adjusted means of the gender awareness post-test.

Post-test	Group	Mean	(SD)	*p*-Value
Public gender consciousness	Experimental	39.72	(0.73)	0.020 *
	Control	37.25	(0.73)	
Private gender consciousness	Experimental	65.78	(1.47)	0.026 *
	Control	61.05	(1.47)	

Experimental group: *n* = 41; control group: *n* = 41; SD: standard deviation; * *p* < 0.05.

**Table 5:** Two-way MANOVA for dependent variable in public gender consciousness.

Source	SS	*df*	MS	*F*	*p*-Value
Between-subjects					
Group	106.613	1	106.613	3.302	0.073
Within + residual	2582.963	80	32.287		
Within-subjects					
Test	803.470	1	803.470	29.794	<0.000 **
Test × group	14.640	1	14.640	0.543	0.463
Test × (within + residual)	2157.390	80	26.967		

SS: sequential sums of square; *df*: degrees of freedom; MS: mean squares; ** *p* < 0.01.

**Table 6:** Two-way MANOVA for dependent variable in private gender consciousness.

Source	SS	*df*	MS	*F*	*p*-Value
Between-subjects					
Group	128.125	1	128.125	2.981	0.088
Within + residual	3437.988	80	42.975		
Within-subjects					
Test	2187.811	1	2187.811	26.860	<0.000 **
Test × group	241.470	1	241.470	2.965	0.089
Test × (within + residual)	6516.220	80	81.453		

SS: sequential sums of square; *df*: degrees of freedom; MS: mean squares; ** *p* < 0.01.

**Table 7:** One-way MANOVA results on the critical thinking pretest.

Pretest	Group	Wilks’ Λ = 0.989 *F* (3, 61)= 0.296, *p*-Value = 0.828		
		Mean	(SD)	*F* (1, 80) (*p*-Value)
Systematicity and analyticity	Experimental	40.17	(6.37)	0.015
	Control	40.34	(6.36)	(0.904)
Inquisitiveness and conversance	Experimental	31.05	(5.88)	0.287
	Control	30.39	(5.23)	(0.594)
Maturity and skepticism	Experimental	26.80	(4.77)	0.359
	Control	26.17	(5.36)	(0.551)

Experimental group: *n* = 41; control group: *n* = 41; SD: standard deviation; *F* (1, 80): tests of between-subjects effects; Wilks’ Λ: Wilks’ lambda; *F* (3, 78): *F* (hypothesis degrees of freedom, error degrees of freedom).

**Table 8:** One-way MANCOVA results on the critical thinking post-test.

**Post-Test**	**Covariance**		
	**Systematicity and Analyticity** **(Pretest)**	**Inquisitiveness and Conversance** **(Pretest)**	**Maturity and Skepticism** **(Pretest)**
	**Wilks’ Λ** ***F* (3, 75)** **(*p*-value)**		
	0.639	0.789	0.752
	14.108	6.699	8.229
	(<0.000 **)	(<0.000 **)	(<0.000 **)
	**Tests of Between-Subjects Effects** ***F* (1, 77)** **(*p*-value)**		
Systematicity and analyticity	8.286	1.160	0.638
	(0.005 **)	(0.285)	(0.427)
Inquisitiveness and conversance	0.766	10.592	0
	(0.384)	(0.002 **)	(0.991)
Maturity and skepticism	2.428	1.908	5.997
	(0.123)	(0.171)	(0.017 *)

Experimental group: *n* = 41; control group: *n* = 41; SD: standard deviation; Wilk’s Λ: Wilks’ lambda; *F* (3, 75): *F* (hypothesis degrees of freedom, error degrees of freedom); * *p* < 0.05; ** *p* < 0.01.

**Table 9:** MANCOVA comparison of the adjusted means of the critical thinking post-test.

Post-Test	Group	Mean	(SD)	*p*-Value
Systematicity and analyticity	Experimental	48.93	(0.98)	0.003 **
	Control	44.73	(0.98)	
Inquisitiveness and conversance	Experimental	37.29	(0.77)	0.012 *
	Control	34.47	(0.77)	
Maturity and skepticism	Experimental	33.14	(0.63)	0.005 **
	Control	30.55	(0.63)	

Experimental group: *n* = 41; control group: *n* = 41; SD: standard deviation; * *p* < 0.05; ** *p* < 0.01.

**Table 10:** Two-way MANOVA for dependent variable in “inquisitiveness and conversance”.

Source	SS	*df*	MS	*F*	*p*-Value
Between-subjects					
Group	146.494	1	146.494	3.622	0.061
Within + residual	3235.366	80	40.442		
Within-subjects					
Test	1091.030	1	1091.030	60.059	<0.000 **
Test × group	62.201	1	62.201	3.424	0.068
Test × (within + residual)	1453.268	80	18.166		

SS: sequential sums of square; *df*: degrees of freedom; MS: mean squares; ** *p* < 0.01.

**Table 11:** Two-way MANOVA for dependent variable in “maturity and scepticism”.

Source	SS	*df*	MS	*F*	*p*-Value
Between-subjects					
Group	128.201	1	128.201	4.711	0.033 *
Within + residual	2176.854	80	27.211		
Within-subjects					
Test	1175.128	1	1175.128	84.115	<0.000 **
Test × group	52.738	1	52.738	3.775	0.056
Test × (within + residual)	1117.634	80	13.970		

SS: SEQUENTIAL sums of square; *df*: degrees of freedom; MS: mean squares; * *p* < 0.05; ** *p* < 0.01.

**Table 12:** Two-way MANOVA for dependent variable in “systematicity and analyticity”.

Source	SS	*df*	MS	*F*	*p*-Value
Between-subjects					
Group	162.006	1	162.006	2.643	0.108
Within + residual	4903.195	80	61.290		
Within-subjects					
Test	1771.470	1	1771.470	70.122	<0.000 **
Test × group	191.030	1	191.030	7.562	0.007 **
Test × (within + residual)	2021.000	80	25.262		

SS: sequential sums of square; *df*: degrees of freedom; MS: mean squares; ** *p* < 0.01.
